# Pathological Interplay of ROS With Myofibroblasts: An Impediment to Corneal Restitution

**DOI:** 10.1155/omcl/5547254

**Published:** 2026-06-15

**Authors:** Mohammad Yahya Karimi, Abasalt Hosseinzadeh Colagar

**Affiliations:** ^1^ Department of Molecular and Cell Biology, Faculty of Basic Science, University of Mazandaran, Babolsar, Iran, umz.ac.ir

**Keywords:** cornea, healing, myofibroblast, pathogenesis, reactive oxygen species

## Abstract

Myofibroblasts are morphologically similar cells with diverse origins that exhibit characteristics of both fibroblasts and smooth muscle cells. Following insults, myofibroblasts play critical roles in tissue reintegration and restitution. However, their prolonged presence and activity impede physiological recovery, leading to persistent or progressive tissue complications, as evidenced in corneal fibrosis and opacification. Reactive oxygen species (ROS) are key signaling intermediates in various cellular events, playing critical roles in the physiology of myofibroblasts. However, when dysregulated, these molecules can engage in misinstructive manners with myofibroblasts, directing these cells toward pathogenic states and behaviors. In brief, dysregulated ROS pathologically modulate myofibroblast differentiation, extracellular matrix (ECM) remodeling, and immune evasion, maintaining self‐perpetuating cycles of myofibroblast survival. The mediation of ROS promotes maladaptive intra and extracellular responses that contribute to myofibroblast persistence by enhancing ECM stiffness, increasing resistance to apoptosis, inducing senescence, and impairing immune clearance. ROS‐mediated alterations in ECM components, most notably in proteoglycans (PGs) and glycosaminoglycans (GAGs), further dysregulate the ECM and make it more conducive to myofibroblast persistence. Additionally, ROS‐induced immune privilege mechanisms prevent the proper clearance of myofibroblasts. Therefore, targeting ROS collectively offers promising therapeutic potential for mitigating their pathological presence and behavior, thereby enhancing overall corneal recovery following insults.

## 1. Introduction

The cornea can be insulted in various ways, but as it recovers, some degree of cellular and structural disarrangements becomes evident. The tissue’s “restitution machinery” is primarily condemned for these sequels, whose primary responsibility is to tailor repair responses according to the insult’s location, magnitude, and type [1]. Typically, severe corneal insults trigger exaggerated restitutive responses that rarely resolve without complications. However, if the insult is milder, the struggle for functional and structural restitution may either conclude flawlessly or result in varying degrees of abnormalities.

Following an insult, the restitution of corneal function, integration, and transparency demands the coordinated actions of multiple cells and factors. However, among these, a group of fibrogenic cells bears the main culprits behind improper tissue restitution. These cells are known as “Myofibroblasts,” and play pivotal roles in physiological repair by generating contractile forces and producing extracellular matrix (ECM) components required for tissue reintegration and remodeling [2].

Myofibroblasts are typically expected to undergo programmed cell death once they have fulfilled their physiological roles [3]. However, they may persist and exhibit activities beyond physiological boundaries [1], as occasionally observed following infections, traumas, or even surgical interventions in the cornea [4]. The unregulated formation and sustained presence of myofibroblasts, even with low‐grade ongoing ECM deposition and remodeling activities, interfere with the physiological repair and exacerbate disharmonies within a recovering tissue [5]. Moreover, the opaque nature of these cells [6, 7], combined with their inhibitory effects on nerve regeneration [8, 9] and structural layer reintegration [2], adds additional objections to their detrimental presence and activity within a recovering cornea.

Despite several efforts to mitigate the detrimental presence and behaviors of myofibroblasts, effective remedies remain elusive. This challenge, perhaps, is due to the diversity of regulatory factors and signaling molecules, which, through their intricate feed‐forward and feedback loops, introduce substantial complexity into the success of therapeutic strategies that target pathological myofibroblasts within a recovering tissue.

Reactive oxygen species (ROS), as a key group of such regulatory factors, orchestrate a variety of physiological processes in recovering tissues. These molecules mediate pivotal actions throughout the lifespan of myofibroblasts, regulating key aspects of their physiology, including activation, transition, development, actions, and fate. However, at dysregulated levels, ROS can be translated adversely by myofibroblasts, perverting these cells to exhibit pathogenic behaviors.

This review intends to highlight the pathological interplay between ROS and the mechanisms that eventually encourage myofibroblasts to remain active in corneal tissue. In this review, we intended to provide a comprehensive understanding of the mechanisms by which ROS influence the behavior of myofibroblasts and contribute to corneal pathology, hoping to be considered while designing therapeutic strategies targeting these pathological entities.

## 2. Structure of the Cornea

The cornea, as the anatomical context of this discussion, is the outermost layer of the eye. The tissue is distinguished by its inherent transparency and curative surface, which together provide significant refractive power to the eye. Due to its avascularity, the cornea relies on surrounding circulating fluids to maintain its homeostasis and metabolic demands [10].

**Figure 1:**
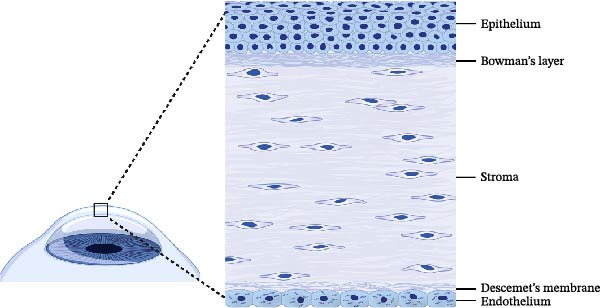
Schematic representation of the corneal layers. This is an original figure created by the authors of the manuscript using Adobe Illustrator.

Structurally, the cornea is composed of five unique layers that support its function (Figure 1). On the outermost surface, five–seven uniform layers of fast‐regenerating epithelial cells, collectively referred to as the epithelium, constantly shed and regenerate through multiplication to preserve the integrity and functionality of the layer. Located just beneath the epithelium, a tough acellular membrane known as Bowman’s layer separates the epithelium from the underlying stroma. The condensed and randomly oriented collagen fibrils of this layer, mainly type I, shape the corneal mechanical structure while providing additional physical protection and biochemical filtration for the adjacent stroma [11]. The stroma, at the center of the cornea, accounts for ~90% of its thickness. The spatial structure of stroma is formed from parallelly arranged thin collagen fibrils, mainly type I (80%–90%), that subsequently construct several thicker layers called lamellae. These lamellae are hydrated matrices rich in glycoproteins, glycosaminoglycans (GAG)‐chained proteoglycans (PGs), and salts, maintained by a small population of interspersed quiescent fibroblasts known as keratocytes [12–14]. Another thin acellular layer of collagen fibrils, mainly type IV, stratifies the posterior side of the stroma and is recognized as Descemet’s membrane. While this layer demonstrates less strength in comparison to the Bowman’s, it effectively serves as the basement membrane for the endothelium. Last, a monolayer of nonproliferative squamous cells, rich in mitochondria, forms a layer and furnishes the innermost surface of the cornea. This layer is known as the endothelium, and the cells of this layer are responsible for the aqueous transport of solutes into the stroma while maintaining its hydration [15–17].

## 3. Myofibroblasts in the Cornea: Physiology and Pathobiology

The cornea, as the outermost layer of the eye, is constantly exposed to potential insults. Those insults can compromise all structural layers of the cornea or be confined to a particular layer. In any circumstances, the tissue’s response is carefully tailored according to the location, type, and severity of the insult. For instance, injuries confined to the epithelium heal quickly through the proliferation of remaining epithelial cells or mobilized limbal stem cells, which reepithelialize the injured area and restore regular biomolecular transport. Further, injuries to the endothelium, though less common, are repaired by the migration and stretching of surviving endothelial cells to refurnish the denuded area. While damages to Bowman’s layer are typically resolved by replacing the damaged area with epithelial cells or scar tissue, the Descemet membrane regenerates successfully through the continuous secretion of its components by endothelial cells. Any failure in the reintegration of the epithelium or endothelium, along with their supporting membranes, results in the unregulated permeation of biomolecules, salts, and water into the stroma, thereby disrupting stromal homeostasis or recovery following insults [1, 15–18].

Stromal repair is more intricate and requires coordinated crosstalk between participating cells, ECM components, growth factors, and inflammatory mediators [19]. This repair is initially triggered by the presence of adequate and sustained levels of promoting factors such as transforming growth factor‐β (TGF‐β) and platelet‐derived growth factor (PDGF). These factors are present only if the insult encompasses epithelium and/or endothelium along with their supportive membranes [1]. In such cases, a narrow acellular area rapidly forms within the afflicted area due to the disappearance of adjacent residual keratocytes. This phenomenon is thought to create a physical barrier of deceased cells, which restricts the infiltration of toxins and pathogens into the stroma. Interestingly, the extent of the damage defines the level of apoptosis in neighboring keratocytes [20], possibly through the degree of interactions between keratocytes’ interleukin (IL)‐1 receptors and IL‐1 released from damaged cells [1].

Over a period of a few hours to several days, an increasing population of bone marrow‐derived cells, including fibrocytes, lymphocytes, macrophages, and monocytes, migrates into the decellularized area. These cells initiate to secrete various cytokines, growth factors, and proteases that are required for the coordinated crosstalk between keratocytes and the ECM. In response, quiescent keratocytes activate and deposit large amounts of fibronectin into the ECM. Concurrently, they express α5β1 integrin receptors on their surfaces [21], enabling them to migrate toward the repair zone on the freshly deposited fibronectin matrices, known as “provisional ECM.” These integrin receptors also modulate keratocyte cytoskeleton reorganizations by allowing the formation of focal adhesions to fibronectin [22]. At this stage, the activated keratocytes exhibit an intermediate phenotype (neither resembles keratocytes nor mature myofibroblasts), sometimes referred to as proto‐myofibroblasts or activated fibroblasts. These cells are highly synthetic and fulfill most tissue restitution requirements, including cellular proliferation (e.g., DNA replication) and ECM demands.

If contractile forces are also demanded (e.g., wound closure), proto‐myofibroblasts undergo further development and acquire a distinct α‐smooth muscle actin phenotype (α‐SMA+). These α‐SMA+ cells are classified as mature myofibroblasts and are distinguished by an abundance of stress fibers and focal adhesions, which enable them to exert contractile forces effectively. Their contractile nature originates from dispersed cytoplasmic bundles of actin and myosin that are strengthened by the incorporation of de novo synthesized α‐SMA proteins into their structures [23, 24]. The presence of α‐SMA is often considered a hallmark of mature myofibroblasts [25] and facilitates the classification of these heterogeneous cells [3]. Mature myofibroblasts are stationary and deposit lower amounts of ECM components compared to proto‐myofibroblasts.

In addition to contractile forces, myofibroblasts are responsible for synthesizing various ECM proteins, including different types of collagens (type I to VI and type XVIII), chondroitin sulfate, decorin, fibronectin, GAGs, heparin sulfate, hyaluronic acid, laminins, nidogen‐1, nidogen‐2, tenascin, and thrombospondin [26, 27]. Moreover, these cells synthesize different matrix metalloproteinase (MMP) enzymes such as MMP‐1, ‐2, ‐3, and ‐9, and their tissue inhibitors (TIMP_S_), enabling them to remodel the ECM at neutral pH [28–31]. Interestingly, these cells also demonstrate pathogen‐eliminating characteristics (e.g., bacteria or viruses) as they express innate immune system receptors such as toll‐like receptors‐2, ‐3, and ‐9 [32, 33].

**Figure 2:**
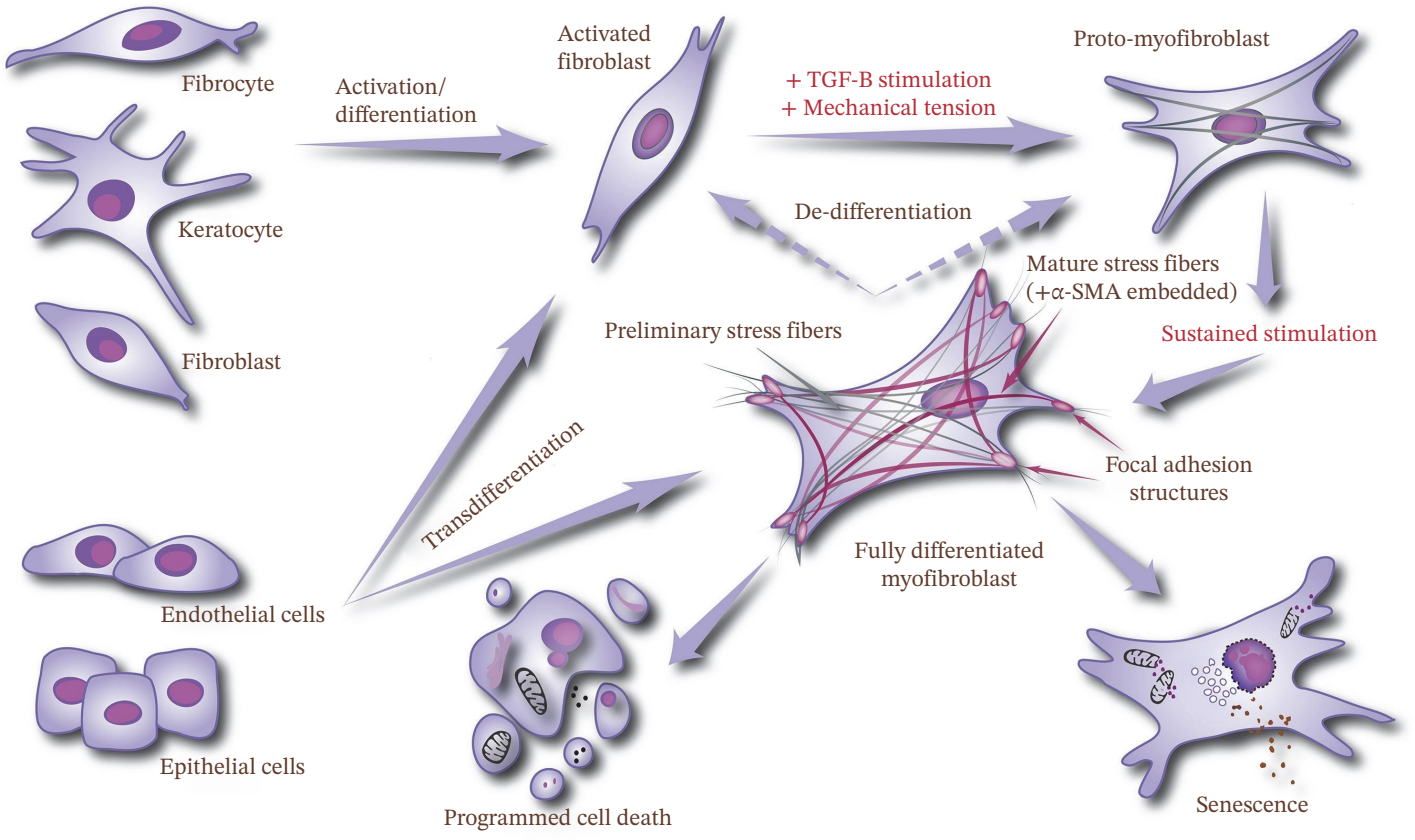
Summarized illustration of myofibroblasts’ life cycle. Various progenitor cells can differentiate into myofibroblasts in the cornea. Keratocytes, fibroblasts, and fibrocytes transit into mature myofibroblasts via a two‐step process, utilizing an intermediate phenotype [34]. In contrast, epithelial and endothelial cells transdifferentiate directly or indirectly through EMT or EndMT phenomena. Several factors trigger the activation, differentiation, or trans‐differentiation of these precursors. However, following the initial transition, sustained TGF‐β1 signaling or increased mechanical tension is required for further development into proto‐myofibroblasts or fully differentiated myofibroblasts. Upon the removal of pro‐survival signals, mature myofibroblasts typically undergo programmed cell death, although they may also acquire a senescent phenotype. Interestingly, some studies have also highlighted the potential for dedifferentiation in cultured myofibroblasts. This is an original figure created by the authors of the manuscript using Adobe Illustrator.

Myofibroblasts originate from multiple precursors in vitro; however, as depicted in Figure 2, the primary precursors of corneal myofibroblasts are keratocytes and bone marrow‐derived fibrocytes [35]. In a mouse model of corneal injury, Lassance et al. [36] reported that 21 days postinitial injury, 30%–50% of αSMA+ cells in the stroma were derived from keratocytes, with the remaining originating from fibrocytes. Additionally, these cells can transdifferentiate from epithelial or endothelial cells through the EMT or EndMT (i.e., epithelial– or endothelial–mesenchymal transition) processes, respectively [37, 38]. However, determining the exact function and degree of contribution of myofibroblasts from different origins in corneal repair remains controversial, as these cells exhibit consistent phenotypical characteristics [39] yet display distinct protein profiles based on their origin [40].

Typically absent in an unwounded cornea [20, 41], the myofibroblasts’ emergence requires adequate and sustained provoking signals [42–44]. Their emergence and maturation can be triggered through various mechanisms and pathways in in vitro settings. In contrast, in vivo they depend on the concurrent influence of biochemical signaling (e.g., TGF‐β) [45] and biomechanical stimulation (e.g., ECM stiffness) [3]. The extent and duration of these signals determine the heterogeneity and size of the myofibroblasts’ population, enabling them to adopt various restitution and justification scenarios [3]. Additionally, the maturation of myofibroblasts appears to be both species‐ and time‐dependent, varying from 1 week to several months [20, 36, 42, 46, 47].

Upon maturation, myofibroblasts can remain indefinitely (months to decades), in line with the persistence of provoking signals [48]. Reduction in the intensity of these requisite signals, below pro‐survival thresholds, triggers intrinsic or extrinsic programmed cell death in myofibroblasts [3]. Their clearance paves the path for stromal keratocytes to migrate into the damaged area to repopulate it and to remodel the repair ECM [49, 50], a process that can persist for months or years [51]. Moreover, myofibroblasts may acquire a senescent phenotype, a profibrotic state that allows them to persist indefinitely, functioning either in tissue improvement [52] or deterioration [53]. Interestingly, in vitro transited myofibroblasts have also demonstrated dedifferentiation potential, an inherent characteristic that allows them to reacquire fibroblastic/quiescent phenotypes [54]; however, the underlying mechanisms and physiological relevance of such phenotypic reversal remain to be fully elucidated.

## 4. Myofibroblasts Persistence in the Cornea: Consequences and Involved Mechanisms

As mentioned, myofibroblasts typically undergo programmed cell death after fulfilling their physiological roles [3]. Their clearance is physiologically secured by various mechanisms, which include ECM softening/degradation, growth factor withdrawal, proapoptotic factors signaling, senescence acquisition, and subsequent immune clearance. However, myofibroblasts can evade these clearance scenarios, thereby persisting beyond physiological boundaries [55].

Based on reports, sustained pro‐survival signaling originating from the ECM, recruited immune cells, or even myofibroblasts themselves are the main mechanisms involved in maintaining their contractile and secretory activities. However, evidence also indicates intrinsic impairment of self‐clearance mechanisms in these cells, with studies reporting acquired apoptosis‐resistant phenotypes (e.g., senescence) by these cells that confer immune evasion. Regardless of the situation, persistence of myofibroblasts leads to their population propagation, encompassing both regular and death‐escaped subtypes [3, 19, 56, 57]. Underlying mechanisms involved in the survival of myofibroblasts, which will be discussed in the relevant sections, are summarized in Table 1.

**Table 1:** List of the primary mechanisms involved in the pathological persistence of myofibroblasts.

Mechanism	Role in myofibroblast persistence	References
Sustained TGF‐β signaling	Promotes transition and survival of myofibroblasts by inducing α‐SMA expression and ECM deposition	[58–60]
Enhanced ECM stiffness	Activates mechano‐transduction pathways, reinforcing myofibroblast contractility, ECM deposition and remodeling activities, and survival	[56, 61–64]
Enhanced apoptosis priming	Promotes resistance to apoptosis in primed for death myofibroblasts through inhibiting proapoptotic pathways	[3, 65–69]
Dysregulated senescence	Regulates apoptosis priming, extracellular trafficking, and senescence in myofibroblasts mainly through SASP propagation and mitochondrial dysfunction	[70, 71]
Dysregulated autophagy	Supports the accumulation of both functional and senescent myofibroblast populations and their ECM deposition and remodeling activities	[72–74]
Immune evasion	Evasion of both regular and senescent myofibroblasts from immune clearance scenarios	[3, 55, 75]
Impaired structural reintegration	Influences stromal remodeling and myofibroblast survival via uncontrolled profibrotic factors extracellular vesicle infiltration	[50, 76, 77]

Sustained presence of myofibroblasts, even with low‐grade ongoing ECM deposition and remodeling activities, interferes with the physiological repair, ultimately resulting in chronic overactivity, sustained tissue contracture, and subsequent hypertrophic scarring [5, 24]. In addition, the opaque nature of these cells [6, 7], combined with their inhibitory effects on nerve regeneration [8, 9] and structural layer reintegration [2], presents further objections to their pathologic presence and behavior within a recovering cornea. Although not directly validated on humans, the experimental findings summarized in Table 2 demonstrate the myofibroblast pathogenic potentials in corneal pathology, indicating the importance of targeting these cells in corneal complications.

**Table 2:** Experimental findings demonstrating potentials of myofibroblasts in the pathology of cornea.

Study	Models	Study findings	Relevance to corneal pathology
Karamichos et al. [78]	Cultured human corneal fibroblasts	Myofibroblasts excrete large quantities of extracellular matrix, including type 1 collagen and fibronectin	Contributes to stromal opacity
Jester et al. [79]	Cultured rabbit corneal keratocytes	Myofibroblasts produce diminished levels of crystallin protein	Decreases corneal transparency
Heindl et al. [80]	Two DMEK human corneal donor	Endothelial‐mesenchymal transition (α‐SMA+) is associated with abnormal posterior fibrillar collagenous layer formation	Interface opacity and graft failure
Okumura et al. [81]	Cultured monkey corneal endothelial cells	In vitro inhibition of EMT decreases collagen I and fibronectin expression and deposition	Compromises corneal transparency
Kawashima et al. [82]	Donor human pannus tissue	Implication of Epithelial‐mesenchymal transited cells (α‐SMA+) in tissue fibrosis	Development of corneal subepithelial fibrosis
Netto et al. [83]	PRK rabbit model	Mitomycin C induces apoptosis in myofibroblasts	Reduced corneal haze
Jeon et al. [8]	PRK cat model and cultured cat corneal keratocytes	Myofibroblasts inhibit neurite outgrowth	Inhibition of corneal nerves regenerating
Bühren et al. [84]	PRK cat model and cultured cat corneal keratocytes	Anti‐TGF‐β treatment reduced myofibroblast formation and decreased nonspherical higher‐order aberrations	Diminished levels of higher order aberrations
MøSller‐Pedersen et al. [85]	PRK rabbit model	Anti‐TGF‐β treatment reduced myofibroblast formation	Reduced corneal haze
Santhanam et al. [86]	PRK rabbit model	Myofibroblasts establishing stacked layers beneath the epithelium, preventing basement recovery	Delayed recovery and prolonged stromal haze
Netto et al. [77] and Mohan et al. [20]	PRK rabbit model	Increased stromal haze is linked to α‐SMA + myofibroblasts density	Development of corneal haze
Milani et al. [87]	EL abrasion rabbit model	Rapamycin Inhibits the formation of myofibroblasts	Inhibition of corneal haze
Gupta et al. [88]	PRK rabbit model	Smad7 overexpression significantly reduced α‐SMA + myofibroblasts	Inhibition of corneal haze
Saika et al. [89]	Alkali‐burn mice model	Smad7‐ adenovirus treatment abolished the generation of myofibroblasts	Suppression stromal ulceration and opacification
Huxlin et al. [90]	LA cat model	Troglitazone (ligand of PPARγ) reduced α‐SMA+ myofibroblasts	Inhibition of corneal haze
Chowdhury et al. [91]	Alkali‐burn rat model and cultured rat corneal fibroblasts	Pirfenidone nanoparticles reduced α‐SMA+ and collagen I expression, both in vitro and in vivo	Reduction of corneal haze

Abbreviations: EL, excimer laser; LA, laser ablation; PRK, photorefractive keratectomy.

In the context of the cornea, opacification and fibrosis are among the significant causes of vision impairment, which typically develop following traumas, infections, or even surgical interventions [4, 19, 92, 93]. While not confirmed by histopathological examinations in the human corneas, experimental evidence strongly suggests the involvement of myofibroblasts in the development of such conditions. Additionally, the well‐established mechanistic link between pathological myofibroblast behaviors and corneal fibrotic outcomes makes these cells the primary culprits for such corneal complications.

However, it should be noted that due to the indispensable roles of myofibroblasts in physiological repair, their activity prevents corneal perforations and—at least partially—preserves ocular integrity. Therefore, to avoid rushed misinterpretations, it is crucial to meticulously determine the pathogenic states of these cells. However, two major obstacles complicate such determinations in recovering corneas: (1) the slow progression of events in the cornea, which predominantly tends to resolve overtime [50], and (2) the inherent heterogeneity of myofibroblasts, which naturally is required to adapt to various healing scenarios. Consequently, despite being effortful and time‐intensive, a long‐term, categorized follow‐up is mandatory to bridge these gaps.

## 5. ROS: Homeostasis and Perturbations in the Cornea

ROS are a substantial family of radical and nonradical derivatives of oxygen (O_2_), whose prominent members include hydrogen peroxide (H_2_O_2_), hydroxyl radicals (⋅OH), peroxyl radicals (ROO⋅), superoxide anions ($\cdot {\text{O}}_{2}^{-}$), and singlet oxygen (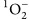). These molecules, ions, or radicals are naturally generated as byproducts of biological processes such as electronic excitation or reduction–oxidation (redox) reactions. Despite their harmful effects on biomolecular structure and function, ROS play critical roles in regulating biological processes as secondary messenger‐signaling intermediates. In a general scheme, the cellular interpretation of ROS levels follows a concentration‐dependent hierarchy: (1) deficient levels induce cell cycle arrest, (2) physiological concentrations maintain regular cellular functions, (3) moderately elevated levels trigger defensive responses, and (4) excessive accumulation hustles cells toward genomic instability, cellular senescence, and ultimately oxidative stress (OS)‐induced cell death [94, 95].

In healthy corneas, endogenous ROS primarily arise from mitochondrial respiration and endoplasmic reticulum protein folding [96, 97] processes of the resident cells, while exogenous ROS result from atmospheric oxygen and ultraviolet radiation (UVR)‐induced ionization. To physiologically maintain ROS, the cornea utilizes a group of specialized enzymatic proteins known as antioxidants, including catalase, glucose‐6‐phosphate dehydrogenase, superoxide dismutase, glutathione peroxidases and reductase, and aldehyde dehydrogenases. These proteins donate their electrons to ROS molecules, preventing these reactive molecules from acquiring electrons from other biomolecules. In addition, the scavenging potential of several nonenzymatic low‐molecular‐weight antioxidants, including albumin, ferritin, ascorbic acid, retinol, glutathione, α‐tocopherol, and nicotinamide adenine dinucleotide phosphate (NADPH) [98–105], also contributes to ROS homeostasis in the cornea.

**Figure 3:**
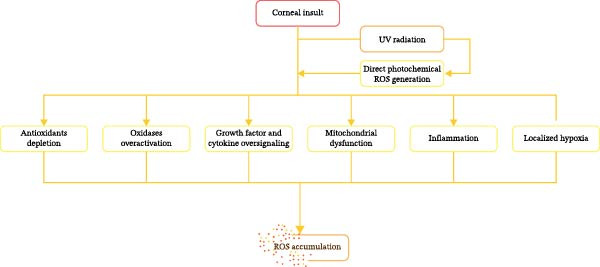
Schematic diagram for sources of ROS perturbations in insulted corneas. Multiple mechanisms contribute to ROS perturbations in an insulted cornea, including antioxidant depletion, overactivity of oxidases, oversignaling of growth factors and cytokines, mitochondrial dysfunction, and sustained inflammation and localized hypoxia. In addition, UV radiation, as a primary external stressor, aggravates ROS accumulation through both photochemical reactions and indirect synergistic intensification of the aforementioned mechanisms.

Although ROS homeostasis is beyond the intentions of this study, its perturbations can occur through multiple mechanisms and pathways (Figure 3). Following insults, the cornea typically exhibits a reduced capacity of both enzymatic and nonenzymatic antioxidants. This antioxidant system impairment allows ROS to accumulate and impact unregulated. For instance, oxidative damage markers such as malondialdehyde and nitrotyrosine increase in insulted corneas, while antioxidant defenses decline [106].

Elevated levels of ROS during corneal insults are often related to increased activity of the oxidases enzymes that generate ROS as an effective measure to implement their roles, including NADPH oxidases (NOXs) [107, 108], xanthine oxidoreductase (XOR), nitric oxide synthase, peroxisomal constituents [109], cytochrome P450, urate oxidase, and D‐amino acid oxidase [110]. Sustained prooxidant activity of these enzymes contributes to the persistence of elevated ROS levels even after the initial phase of recovery. Notably, the family of NOXs (NOX 1–5 and DUOX1/2), particularly NOX2 and NOX4, are the remarkable members whose primary biological actions are heavily involved in the formation and differentiation of myofibroblasts in various organs [111–115]. Furthermore, growth factors and cytokines involved in recovery proceedings can significantly amplify ROS production through mitogenic stimulation [116–120]. Key mediators include TGF, PDGF, fibroblast growth factors (FGF‐1 and ‐2), insulin‐like growth factors (IGFs), hepatocyte growth factor (HGF), keratinocyte growth factor (KGF), ILs‐1, ‐6, and ‐10, and tumor necrosis factor‐α (TNF‐α).

Inflammation and inflammatory responses can also exacerbate ROS accumulation in insulted corneas. The activation and recruitment of immune cells, such as neutrophils and macrophages, further enhance ROS accumulation as a part of their defense mechanisms. The immune system primarily maintains ROS for combating pathogens and regulating the diverse functioning of immune cells; however, this can also lead to collateral damage to the corneal architecture. Concomitantly, ROS amplify the release of pro‐inflammatory cytokines such as IL‐1, TNF‐α, and IL‐6. These cytokines further enhance the activity of ROS‐generating enzymes such as NOXs, creating a vicious cycle that accumulates ROS in the cornea. In addition, through the activation of signaling pathways such as the p38 MAPK and NF‐κB pathways, ROS production is sustained, further amplifying inflammation and cellular dysfunction [106].

Hypoxia is another mechanism that increases ROS in the insulted cornea. Typically, structural disruption and metabolic demands create spatiotemporal oxygen gradients, resulting in localized hypoxia in certain areas of the cornea. While physiological hypoxia orchestrates recovery proceedings through ROS‐mediated regulation of cell migration and proliferation, sustained hypoxia paradoxically exacerbates ROS production via mitochondrial dysfunction and NOX activation [121, 122].

External stressors, such as UV radiation, further exacerbate ROS accumulation in insulted corneas. In compromised corneal structures, UV radiation freely contributes to ROS generation through both direct and indirect mechanisms. UV radiation, both UVA and UVB, directly increases ROS via photochemical reactions, including the excitation of endogenous chromophores (e.g., NADPH) and the direct photooxidation of biomolecules like DNA, lipids, and proteins [123, 124]. These direct UV‐induced ROS act synergistically with pathology‐induced ROS and potentiate other mechanisms involved, including antioxidant depletion, prooxidant enzyme activation, and signaling by growth factors and inflammatory cytokines. By inducing damage to mitochondrial DNA and membranes, UV radiation also exacerbates mitochondrial dysfunction, a well‐known source of cellular ROS [125]. Ultimately, these ROS also intensify inflammatory responses and, by inducing structural disruption and increasing metabolic demand, indirectly promote localized hypoxia [126, 127]. It is worth noting that the cornea’s epithelium and stroma filter the UVC (190–290 nm) and UVB (290–320 nm), respectively, while the lens is mainly responsible for filtering UVA (320–400 nm) [7].

Collectively, ROS accumulation can occur at virtually every stage of corneal repair. When unregulated, they either directly damage the biomolecules or organelles or dysregulate cellular functions through stress‐sensitive pathways. Regardless of the circumstances, ROS pathogenicity is always characterized by a condition typically known as OS, in which ROS levels exceed the antioxidative defense capacity of a living complex, providing a window for them to implement their detrimental effects freely. Oxidative damage is routinely observed in corneal diseases and tragedies [128–130], indicating the importance of targeting ROS in these circumstances.

## 6. ROS–Myofibroblast Interplay and Pathogenicity

To acknowledge the role of ROS in the pathophysiology of myofibroblasts, a comprehensive understanding of their physiological roles is required. ROS mediate distinct physiological actions throughout the lifespan of myofibroblasts, including their activation, differentiation, homeostasis, and fate. While these actions have been well‐characterized in other tissues [95, 131], their specific validation in corneal compartments remains incomplete. Therefore, what follows is a summary of experimental findings from both in vitro and in vivo studies that mechanistically link ROS to myofibroblast presence and behavioral patterns across tissues (Table 3). Accordingly, in the following sections, we will specifically focus on the detailed interplay between ROS and the mechanisms involved in myofibroblast pathology, as outlined in Table 1. This targeted exploration aims to further expand our understanding of how ROS can misinstruct myofibroblasts and perpetuate their physiology, ultimately contributing to the corneal pathology.

**Table 3:** Experimental evidences demonstrating the potential of ROS in myofibroblast presence and behavior.

Study	Model	Key findings	Relevance to myofibroblasts physiology
Formation and ECM production			
Hecker et al. [111]	Primary lung mesenchymal cells from IPF patients	TGF‐β‐induced myofibroblast differentiation is mediated by ROS, which activate Smad 3	ROS mediate canonical TGF‐β/Smad signaling pathway in myofibroblast differentiation
Bocchino et al. [132]	Primary lung fibroblasts from IPF patients	Fibroblasts differentiate into myofibroblasts with high ROS levels from an NADPH oxidase system; ROS scavenging reverses the phenotype	ROS are required for myofibroblasts differentiation
Jain et al. [133]	Human lung fibroblasts	Inhibition of mitochondrial ROS attenuates TGF‐β‐induced gene expression	Mitochondrial ROS regulate myofibroblast transition.
Taddei et al. [134]	Human fibroblasts with mitochondrial complex I dysfunction	Mitochondrial dysfunction leads to ROS overproduction, which correlates directly with myofibroblast activation, and ECM remodeling	Mitochondrial ROS regulate myofibroblast transition.
Bindu et al. [135]	Human lung fibroblasts	SIRT3 deficiency increases mitochondrial ROS, enhancing TGF‐β‐induced differentiation	Mitochondrial ROS control sensitivity to TGF‐β and myofibroblast transition
Kim et al. [136]	Retinal pigment epithelial	TGF‐β induced ROS drives EMT via ERK1/2‐mTORC1‐NOX4 axis	ROS mediate TGF‐β‐induced EMT
Rhyu et al. [137]	Rat proximal tubular epithelial cells	Antioxidants effectively inhibited TGF‐β‐induced cellular ROS and EMT	ROS mediate TGF‐β‐induced EMT
Giordo et al. [138]	Human Retinal Endothelial Cells	Resveratrol counteracts NOX‐mediated endothelial to mesenchymal transition	ROS mediate TGF‐β‐induced EndMT
Brown et al. [115]	Rabbit conjunctival fibroblasts	TGF‐β induces NOX4 expression; NOX4‐dependent ROS is required for collagen production. Targeting NOX4 inhibits this effect	ROS are required for ECM synthesis and contractile function of myofibroblasts
Sugiura et al. [139]	Human fetal lung fibroblasts	N‐acetylcysteine (ROS scavenger) inhibits TGF‐β‐induced α‐SMA and fibronectin secretion	ROS scavenging prevents differentiation and ECM synthesis.
Lijnen et al. [140]	Rat cardiac fibroblasts	Inhibition of superoxide dismutase induces collagen production in angiotensin II‐treated cells	ROS are required for ECM synthesis by myofibroblasts
Huo et al. [141]	Human corneal epithelial cells	EGF‐stimulated cell adhesion or migration was greatly suppressed in the presence of N‐acetylcysteine	ROS mediate migration and pheno‐conversion of precursors EMT
Lin et al. [142]	Human gingival fibroblasts	Galectin‐1 induced myofibroblast activation, migration and proliferation by triggering ROS production	ROS mediate precursors migration, proliferation and transition
Yang et al. [143]	Human corneal fibroblasts	TGF‐β induces TRPV1 transactivation through SMAD2‐mediated ROS in myofibroblasts, resulting in a positive feedback loop that greatly augments the persistence of activated states for p38 and SMAD2	ROS mediate myofibroblasts survival
Senescence and apoptosis			
Feng et al. [144]	Human hypertrophic scar myofibroblasts	Elesclomol induces excessive intracellular ROS, leading to targeted apoptosis of myofibroblasts via activation of caspase‐3 and release of cytochrome c	ROS mediate apoptosis in myofibroblasts
Borkham‐Kamphorst et al. [145]	Primary portal myofibroblasts	CCN1/CYR61 overexpression leads to ROS production, which dose‐dependently induces both apoptosis and senescence	ROS regulate apoptosis and senescence in myofibroblasts
Larochelle et al. [146]	Human dermal normal wound myofibroblasts and hypertrophic scar myofibroblasts	ROS induce cell death in regular and hypertrophic scar myofibroblasts with different sensitivity	ROS are important mediators of apoptosis in myofibroblasts
Maus et al. [147]	Primary mouse embryo fibroblasts, and nonimmortalized human lung fibroblasts	Senescence induced by iron in these cells and ROS scavengers prevented the emergence of SA‐β‐GAL‐positive cells	ROS regulate senescence in myofibroblasts
Shi et al. [148]	Human scar fibroblasts	Dioscin regulate apoptosis and ferroptosis of scar fibroblasts through mitochondrial ROS	ROS regulate apoptosis in myofibroblasts

Abbreviations: EMT, epithelial–mesenchymal transition; EndMT, endothelial–mesenchymal transition; IPF, idiopathic pulmonary fibrosis.

### 6.1. ROS and TGF‐β‐Based Survival of Myofibroblasts

Typically, effective corneal recovery is coordinated by an array of cytokines and growth factors, including PDGF, EGF, TGF‐β, FGF, TNF‐α, KGF, HGF, IGF, vascular endothelial growth factor (VEGF), and several ILs (IL‐1, ‐6, ‐8) [149–151]. Among these, PDGF and, more prominently, TGF‐β act as major regulators of myofibroblast emergence and survival. PDGF supports the mobilization of myofibroblast precursors, whereas TGF‐β is central to the activation and differentiation of myofibroblasts [57]. Of the three existing isoforms of TGF‐β (i.e., TGF‐β1, ‐β2, and ‐β3), TGF‐β1 (hereafter referred to as TGF‐β) is the key mediator of myofibroblast transition. In conjunction with TGF‐β1, TGF‐β2 is responsible for the excessive ECM synthesis and secretion by these cells [152, 153].

Thus, although TGF‐β is essential for the emergence and activities of myofibroblasts, persistent signaling of this pathway can lead to pathological survival of myofibroblasts in insulted corneas [58, 59], as it enhances ECM production and resistance to apoptosis through the activation of pathways such as FAK‐PI_3_K/AKT and p38 MAPK and suppression of both extrinsic and intrinsic apoptosis [3, 55, 154]. While the detailed mechanisms underlying these pathways fall beyond the scope of this study, key aspects of these mechanisms are detailed in relevant sections.

Following corneal insults, TGF‐β mainly gains access to the stroma through compromised epithelial or endothelial basement membranes. While TGF‐β is initially derived from the tear film, aqueous humor, and epithelial cells [49, 155], it is also synthesized by inflammatory cells, fibroblasts, and myofibroblasts independently as recovery proceeds. Therefore, a sustained reservoir of TGF‐β accumulates in the ECM, establishing both autocrine and paracrine signaling loops that support myofibroblasts’ survival even after the reappearance of the structural barriers [149, 156–159].

The released form of TGF‐β is inactive, complexed with latent TGF‐β binding protein (LTBP) and latency‐associated peptide (LAP), forming a large complex bound to ECM [158]. Upon dissociation by proteolytic cleavage or biomechanical cues [160–162], the activated TGF‐β interacts with its receptors and induces the expression of various genes through both (mothers against decapentaplegic homolog [SMAD])‐dependent or ‐independent pathways [163]. In myofibroblasts, TGF‐β‐mediated transcription of genes associated with collagen deposition and stress fiber formation occurs through receptor‐mediated phosphorylation of SMAD‐2 and ‐3, which, further in complex with SMAD‐4, regulate the transcription of related genes in the nucleus [34, 162].

A key mechanism supporting myofibroblast survival is the reciprocal interplay between TGF‐β and ROS. TGF‐β signaling significantly enhances ROS production in myofibroblasts by upregulating the expression of NOX enzymes [164], specifically NOX2 and NOX4 [165]. Both the canonical (SMAD‐dependent) and noncanonical (PI3K‐mediated) TGF‐β pathways [166] are involved in NOXs expression, and the enzymatic activity is essential for TGF‐β–induced myofibroblast differentiation [165, 167]. Simultaneously, TGF‐β suppresses the expression of various antioxidant enzymes and their corresponding proteins, including glutaredoxin, glutathione peroxidase, glutathione S‐transferase, catalase, and superoxide dismutase [168–174], thereby creating permissive conditions for ROS accumulation.

**Figure 4:**
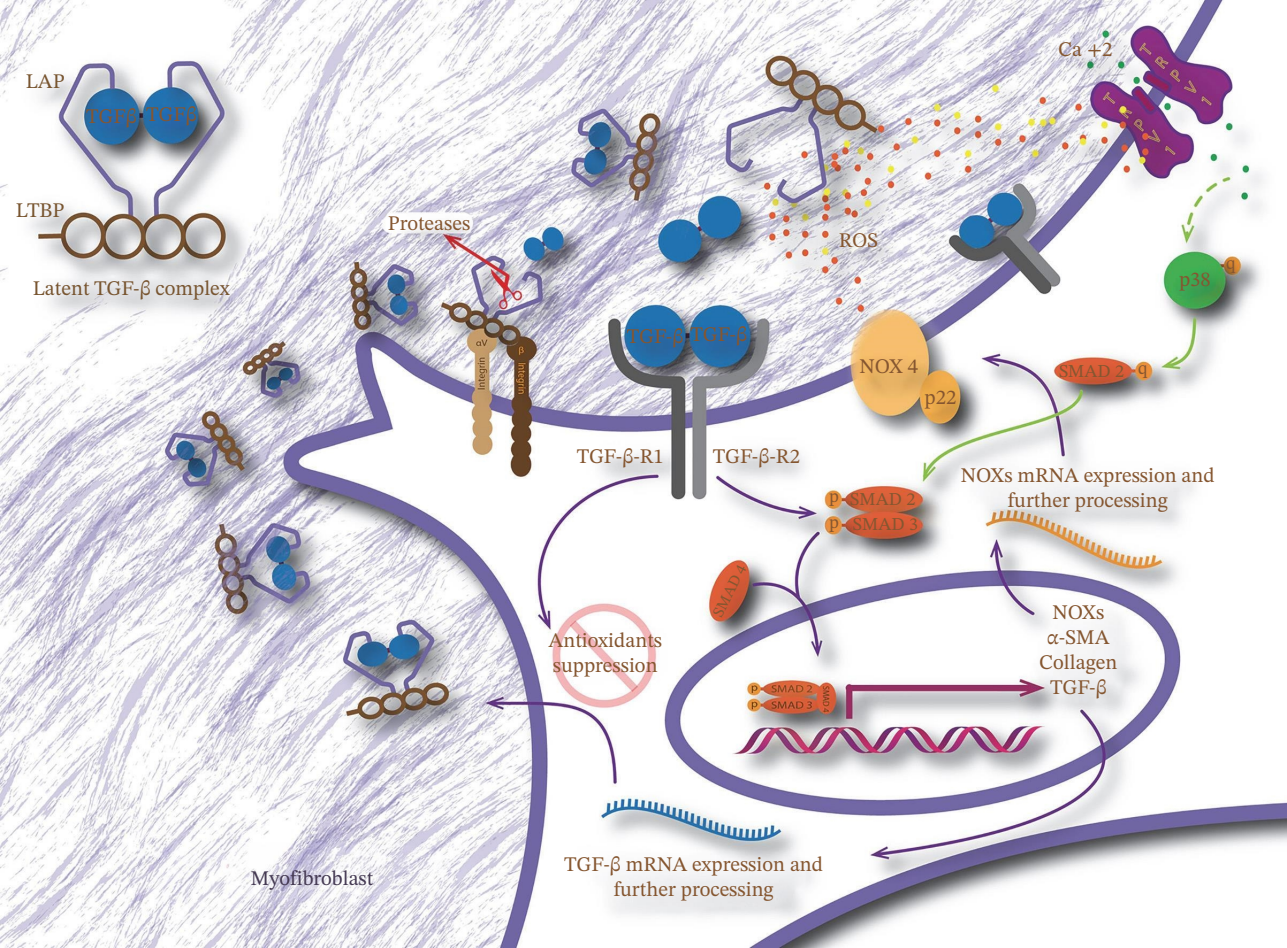
Concise depiction of ROS interplay with biochemical (TGF‐β) signaling in myofibroblasts. The TGF‐β is synthesized in a latent form complexed with latency‐associated peptide (LAP) and latent TGF‐β‐binding protein (LTBP). The secreted complex binds to the ECM, and mechanical stretching of the stiff ECM releases TGF‐β from the complex, allowing the free TGF‐β to bind to its receptors. ROS can also release TGF‐β from its latent complex. This situation creates an uncontrolled, recurrent loop that eventually leads to escalated TGF‐β secretion and activation, as well as subsequent ROS formation. In addition, ROS‐activated TRPV1 further stimulates p38, creating a positive feedback mechanism that sustains SMAD‐2 phosphorylation and ROS production. This is an original figure created by the authors of the manuscript using Adobe Illustrator.

Conversely, ROS directly activate latent TGF‐β by oxidizing critical cysteine residues in LAP. This oxidative modification allows a conformational change in the LAP peptide, which ends with the release of TGF‐β from its latent complex [166]. Notably, this mode of ROS‐based TGF‐β bioactivation exhibits isoform specificity and is restricted to TGF‐β1 [175–177]. Therefore, these interlinked events create a recurrent loop that amplifies ROS production via TGF‐β, further propagating TGF‐β secretion and bioactivation, as depicted in Figure 4.

Moreover, TGF‐β–induced ROS enhance myofibroblast survival through the p38 MAPK pathway. This process highly depends on a self‐amplifying loop where TGF‐β‐induced SMAD‐2 phosphorylation generates ROS, which subsequently activates the transient receptor potential vanilloid 1 (TRPV1) channel. TRPV1 activation further leads to calcium influx and p38 MAPK activation, which in turn enhances SMAD‐2 phosphorylation. This situation creates a seemingly endless cycle where ROS‐activated TRPV1 perpetuates its activation through sustained ROS generation [143, 178]. The ROS that activate TRPV1 are generated through NOX enzymes [179] and mitochondria [180], though their relative contributions remain unclear. Whatever the source, ROS directly activate TRPV1 through oxidative modification of cysteine residues within the channel protein [181]. Concomitantly, the direct inactivation of MAPK phosphatases by ROS further secures this mode of signaling [182], promoting the constant survival of myofibroblasts as long as ROS are present.

Collectively, these interactions establish a self‐perpetuating cycle between TGF‐β signaling and ROS production, ultimately driving the pathological persistence of myofibroblasts in corneal repair. The reciprocal activation of TGF‐β and ROS——through both canonical (SMAD) and noncanonical (p38MAPK/TRPV1) pathways—creates a pathological feedback loop that ultimately sustains myofibroblast survival. Therefore, breaking these vicious cycles by targeting ROS presents a promising therapeutic strategy to mitigate pathological impacts of myofibroblasts and promote corneal recovery.

### 6.2. ROS and ECM‐Based Survival of Myofibroblasts

The ECM is a dynamic and structurally complex network of proteins and polysaccharides that sustains tissue integrity while actively regulating the physiology and behavior of resident cells [183, 184]. In the cornea, fibroblasts respond to ECM biomechanical dynamics by altering cytoskeletal arrangements, expressing α‐SMA, and synthesizing new collagen, all critical steps in their transition to myofibroblasts. Reciprocally, changes induced by these cells on the ECM, particularly regarding stiffness, modulate their further activation, survival, and functional contributions to recovery proceedings [56, 62, 161, 185–188].

Myofibroblasts display both cell–cell junctions known as “fibronexus junctions” and cell—ECM junctions called “focal adhesions” (FAs) [189]. These large macromolecular complexes comprise several components, including integrins, talin, vinculin, paxillin, FAK, and Src, that collectively serve as a unique mechanosensitive system to translate extracellular mechanical signals into intracellular biochemical responses. Through integrin‐based stiffness perception mechanisms, FA complexes enable myofibroblasts to precisely regulate their developmental, remodeling, and compensational activities in response to ECM dynamics [190, 191]. While this system adjusts cellular responses and maturation to the viscoelastic properties of the ECM, it also ensures, in time, induction of apoptosis upon termination of mechanical stimulation. Thereby, the system promotes physiological clearance upon restoration of tissue architecture and tensile strength. However, persistent stiffened ECM‐derived signals allow myofibroblasts to evade apoptosis and persist indefinitely [3, 61, 93].

During tissue recovery, myofibroblasts generate a distinct ECM around themselves. This ECM exhibits reduced elasticity and heightened stiffness compared to natural ECM, which facilitates myofibroblasts to exert their contractile forces effectively [61, 192]. This stiffness is further exacerbated by the intrinsic contractility of myofibroblasts [193] and their ongoing ECM deposition and remodeling activities [2]. Since myofibroblasts augment the stiffness of ECM, and the stiffened ECM, in turn, regulates their activation, differentiation, survival, and activities [194, 195], a recurring cycle is established through these events. Although this cycle initially appears advantageous for maintaining a myofibroblast reservoir during recovery [196, 197], excessive or prolonged signaling through this cycle misinstructs myofibroblasts to persist long after they are no longer physiologically required.

According to reports, prolonged ECM deposition and remodeling alter tissue architecture and mechanics, impairing proper restitution—a phenomenon observed in corneal morphogenesis during prolonged phases of repair and remodeling [45, 51]. Myofibroblasts deposit excessive amounts of ECM components into the corneal stroma, collectively contributing to stromal disorganization and opacity [20, 198, 199]. Excessive ECM deposition also fosters the development of fibrosis and neovascularization in the cornea [200, 201]. Conversely, similar pathology is observed in situations where ECM degradation and turnover are dysregulated [63]. These findings demonstrate the significance of ECM regulations in the pathology of recovering corneas.

Among the ECM components, collagen is the primary determinant of ECM stiffness, where both increased deposition and impaired turnover [63] closely correlated with persistent myofibroblast presence [202]. Proteomic analyses identify collagen I and III as predominant myofibroblast‐derived ECM components in vitro [40], mainly regulated through the TGF‐β/SMAD signaling pathway. Activation of TGF‐β/SMAD upregulates transcription of collagen genes, including COL1A1 and COL1A2 (encoding collagen type I) and COL3A1 (encoding collagen type III) [203].

TGF‐β and ROS cooperatively upregulate collagen synthesis and secretion by myofibroblasts [204]. Therefore, the reciprocal activation of TGF‐β and ROS again creates a reinforcing feedback loop that supports fibrotic ECM accumulation and matrix stiffening. This pathological loop appears to be a promising target as ROS scavengers like N‐acetylcysteine (NAC) potentially disrupt the loop and suppress collagen production [205].

Concurrently, ROS indirectly can sustain ECM stiffness by prolonging FAK pathway activity, a crucial signaling pathway involved in regulating collagen synthesis by myofibroblasts [64, 206]. FAK is a nonreceptor protein tyrosine kinase (PTK) that mediates the signaling through FA complexes. Upon mechanical stimulation, FAK activates and both enhances the expression of collagens [207] and regulates MMPs and their inhibitors (TIMPs), thereby balancing ECM production and degradation [208]. It has been reported that oxidative inactivation of protein tyrosine phosphatases (PTPs) such as LMW‐PTP reversibly promotes FAK signaling, resulting in persistent profibrotic FAK activities. This feedback ensures that the FAK‐mediated ECM synthesis and remodeling remain persistently active in the microenvironments where ROS are present constantly [209, 210].

Besides collagen deposition, dysregulated cross‐linkage of collagen fibers can augment ECM stiffness, creating a more fibrotic microenvironment [211]. While UV radiation can participate in such reactions at reduced paces [212], the process is primarily mediated by the lysyl oxidase (LOX) family of enzymes in the insulted corneas [213]. Typically, LOX enzymes catalyze the covalent cross‐linking of collagen fibers. The oxidative deamidation of reactive aldehyde groups in lysine and hydroxylysine residues of collagen fibers by LOXs makes these fibers insoluble and stiffer, thereby increasing ECM tensile strength and resistance to proteolytic degradation [214, 215]. Upregulation of LOXs is frequently observed during tissue recovery and fibrosis, and the enzyme’s activity is considered a key event in abnormal collagen cross‐linking and haze formation in the cornea [216–218].

Excessive LOX activity enhances collagen cross‐linking, making the ECM more rigid. Enhanced cross‐linking by LOXs further reduces the susceptibility of collagen fibers to proteolytic degradation and promotes their accumulation within tissues [219, 220]. In pathological states, ROS upregulate the expression and activities of LOXs through stimulation of various signaling pathways, including MAPKs, TGF‐β/SMAD, and hypoxia‐inducible factor‐1 (HIF‐1α) [221]. LOX enzymes, in turn, generate ROS as a byproduct of their catalytic activity. Therefore, the combined action of ROS and LOX results in pronounced collagen cross‐linking and sustained ECM stiffening.

Interestingly, ROS present a dualistic influence on collagen cross‐linking. Through oxidation of critical residues in the enzyme’s active site, ROS impair the enzyme’s catalytic function. Also, by directly oxidizing lysine and hydroxylysine residues in collagen fibers, ROS prevent their participation in LOX‐mediated cross‐linking [222]. Consequently, this overall reduction in cross‐linking formation compromises collagen fibril stability and weakens stromal tensile strength. Importantly, this paradoxical effect is not typical of fibrotic conditions, but somewhat relevant to other corneal pathologies such as keratoconus, where diminished collagen deposition and cross‐linking contribute to characteristic structural instability and progressive thinning [223].

Besides collagen regulations and dynamics, ROS also significantly impact GAGs and PGs, which are essential for corneal hydration and biomechanical homeostasis. As stated earlier, ROS cannot independently initiate collagen laydown in fibroblasts [41]; instead, they significantly increase GAG production [224], a key component of PGs. GAGs are highly hydrophilic carbohydrates that function as shock absorbers and viscosity regulators in tissues [225]. Moreover, GAGs also tether growth factors and regulate their release and activation in a controlled manner [226], thereby playing critical roles in physiological recovery. However, uncontrolled secretion and accumulation of GAGs lead to multiple complications in recovering tissues.

Primarily, the GAGs’ strong water‐absorbing capacities cause tissue swelling. Additionally, their excessive accumulation disrupts the regular organization of the ECM that adversely affects cell behavior, tissue mechanics, and signaling pathways. Besides, GAGs can function as damage‐associated molecular patterns (DAMPs) and trigger undesired inflammatory responses [227–229]. Together, these sequelae resemble pathological situations that occur in fibrosis, which are more supportive of myofibroblast persistence.

Besides GAGs’ secretion, ROS also implement direct structural alterations in GAGs. Structurally, GAGs form the backbone of PGs, crucial ECM components that maintain tissue organization and function. Physiologically, PGs perform critical actions in the corneal stroma by facilitating the development of collagen fibrils, maintaining hydration levels, keratocyte proliferation, and intervening in the effects of growth factors [230].

To establish natural architecture, PGs interact with deposited collagen fibers at specific points to shape their diametric uniformity, resembling an envelope structure. These interactions occur through GAG chains that are covalently attached to the core proteins of PGs [231]. ROS can directly cleave these covalent bonds of GAGs, permitting them to dissociate from PGs. This cleavage increases the susceptibility of PGs to enzymatic degradation by proteases, which ultimately leads to collagen fiber aggregation, tissue stiffening, and unregulated growth factor activation and signaling [232, 233]. The degradation and reduction of PGs, combined with increased MMPs activity in recovering tissues, create a stiffer ECM that is more conducive to the persistence of myofibroblasts. In the cornea, UV radiation‐induced ROS also contributes to such fibrillar aggregation, albeit at reduced rates in healthy corneas [97], while remaining more pronounced in recovering corneas where ROS levels are elevated and antioxidant defenses are diminished.

In summary, the dynamic interplay between the ECM and myofibroblasts plays a pivotal role in corneal recovery. The recovering ECM not only provides structural support for myofibroblasts actions, but it also actively regulates the physiology of myofibroblasts. However, several ROS‐mediated events can dysregulate this equilibrium. Excessive ECM deposition, cross‐linking, and remodeling, driven by dysregulated ROS, lead to pathological stiffening, stromal disorganization, and opacity. Furthermore, changes induced by ROS in GAGs and PGs exacerbate these ECM irregularities, promoting edema, inflammation, and the abnormal activation of growth factor pathways.

**Figure 5:**
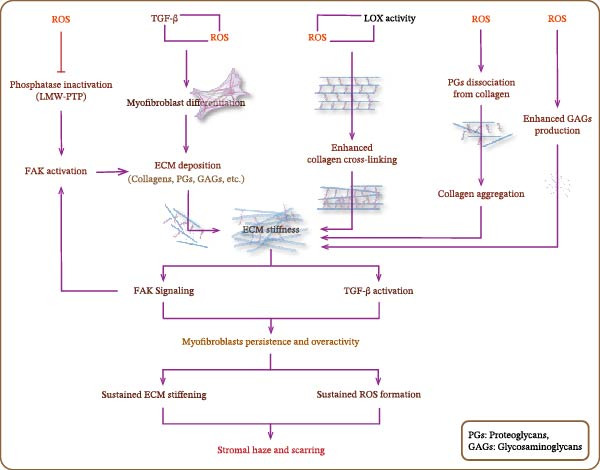
ROS‐mediated mechanisms of pathological ECM stiffness and myofibroblast survival. This is an original figure created by the authors of the manuscript using Adobe Illustrator.

While these ROS‐mediated mechanisms sustain myofibroblast persistence, they also present potential therapeutic targets for intervention. As the key drivers of these mechanisms (Figure 5), ROS are a logical therapeutic target. Therefore, ROS‐based intervention in these vicious cycles can prevent progressive scarring and improve recovery outcomes following corneal insults.

### 6.3. ROS and Enhanced Apoptosis Priming of Myofibroblasts

As mentioned, sustained TGF‐β signaling combined with persistent biomechanical stress can prolong myofibroblast survival [56, 62], leading to pathological tissue remodeling and scarring. However, this survival is neither indefinite nor exclusively maintained by TGF‐β and mechanical cues alone. Instead, it depends on a dynamic equilibrium between multiple integrated factors that collectively determine the fate of myofibroblasts, including signaling networks, metabolic adaptations, and microenvironmental cues [3, 234]. Thus, the fate of myofibroblasts is determined by a functional “threshold” set by the balance of sustained survival signaling versus proapoptotic cues.

In myofibroblasts, the overall balance of this threshold relies on multiple mechanisms, including pro‐survival signals (e.g., TGF‐β, FAK, and antiapoptotic proteins), metabolic adaptation, and external cues from the immune environment and matrix [235–238]. Disruption of these mechanisms can unbalance the threshold, leading to the elimination of myofibroblasts. Therefore, myofibroblast persistence is inherently limited in the absence of continued reinforcement [3, 39, 239]. However, while apoptosis priming enhances the survival thresholds of myofibroblasts, this adaptation also elevates their resistance capacities in stressed conditions [3, 65, 66].

ROS can critically influence this fate‐determining balance [95, 240]. While classically ROS are linked to proapoptotic signaling [240], their role within the fibrotic microenvironment is multifaceted and context‐dependent [94, 129, 241]. Emerging evidence suggests that ROS can contribute to both the *priming for* and the *evasion* of apoptosis that paradoxically determine whether myofibroblasts persist or resolve [65, 107, 166, 234, 242].

Elevated levels of ROS, particularly mitochondrial ROS, typically activate the intrinsic apoptotic pathway [243, 244]. Briefly, by damaging mitochondrial membranes and components, ROS increase mitochondrial outer membrane permeability (MOMP) and trigger the release of cytochrome c. This occurs primarily through ROS‐mediated cardiolipin oxidation, which enables pore formation by oligomerization of BAX and BAK [245–247]. Concurrently, ROS stimulates proapoptotic BH3‐only proteins (including BIM and PUMA) via both direct oxidation and indirect activation through stress kinase pathways such as JNK and p38 MAPK [65, 237, 240, 248]. ROS also oxidatively inactivate particular members of the antiapoptotic BCL‐2 family (i.e., BCL‐2 and BCL‐X_L_), thereby reducing their capacity to neutralize proapoptotic effectors like BIM [240, 249, 250]. Finally, ROS directly activate apoptosis‐executive caspases while simultaneously inhibiting their inhibitors, such as XIAP [251–253].

Numerous reports have demonstrated that ROS scavengers (e.g., NAC) promote myofibroblast survival in vitro when exposed to various stressors, while ROS inducers trigger apoptosis [144, 254–256]. In vivo, antioxidants can sometimes ameliorate fibrosis, partly attributed to reduced myofibroblast apoptosis resistance [257, 258].

However, ROS also act as secondary messengers downstream of TGF‐β, PDGF, and integrin engagement [107, 111]. They can also transiently inhibit PTPs, such as PTP1B, LMW‐PTP, and PTEN, by oxidizing their catalytic cysteines [259–261]. While ROS can directly activate specific kinases, such as Src [154, 262, 263], ROS‐mediated inhibition of PTPs sustains the activation of pro‐survival kinases, including FAK, Src, PI_3_K, and Akt [260, 264–266]. Sustained PI_3_K/Akt activation phosphorylates and inactivates proapoptotic proteins (BAD and FOXO transcription factors) and promotes degradation of others (BIM), while enhancing NF‐κB survival signaling [267–269].

ROS can activate transcription factors (NF‐κB, AP‐1, and HIF‐1α) and co‐activators (YAP/TAZ) that drive expression of antiapoptotic genes (BCL‐2, BCL‐X_L_, XIAP, Survivin) and profibrotic genes [270–272]. YAP/TAZ nuclear translocation, influenced by ROS‐modulated cytoskeletal dynamics, is a key pro‐survival and profibrotic signal [273–275].

Chronically, ROS can also induce adaptive responses in myofibroblasts, upregulating endogenous antioxidants (e.g., glutathione, SOD, and catalase) and stress response proteins (e.g., HO‐1 and Nrf2 targets), enhancing their resistance to OS‐induced apoptosis [240, 276, 277].

Studies show that specific ROS sources (e.g., NOX4 downstream of TGF‐β) are essential for myofibroblast differentiation and survival [107, 166, 278]. Inhibition of ROS‐generating enzymes can paradoxically reduce myofibroblast numbers by inducing apoptosis in some models [279, 280]. Conversely, constitutive activation of pro‐survival pathways, such as Akt and Nrf2, can override ROS‐induced apoptosis signals [267, 269, 280, 281].

Based on reports, myofibroblasts on stiff matrices exhibit heightened mitochondrial priming. These cells express elevated levels of the proapoptotic BH3‐only activator protein BIM [65, 66], potentially exacerbated by mitochondrial ROS. Expression of BIM predisposes myofibroblasts to apoptotic thresholds. However, this primed state is counteracted by robust, ROS‐sustained pro‐survival signaling (FAK–PI_3_K/Akt–YAP/TAZ–BCL‐X_L_ axis) [3, 55, 65, 66, 282, 283]. Therefore, the persistence of myofibroblasts relies on the continuous activity of these survival pathways, which ROS actively potentiate. Termination of survival signals (e.g., ECM softening and growth factor withdrawal) tips the balance, allowing ROS‐mediated proapoptotic mechanisms to execute cell death.

In parallel, microRNAs such as miR‐21 also regulate apoptosis thresholds in matrix‐primed myofibroblasts by coordinating the regulation of survival determinants. By upregulating antiapoptotic BCL‐2 and suppressing both proapoptotic BAX and antioxidants such as superoxide dismutase [284–289], these microRNAs create a paradoxical cellular state in which apoptosis‐primed myofibroblasts maintain their viability through enhanced BCL‐2 expression and compromised ROS clearance.

Within these situations, mitochondrial ROS further solidifies myofibroblast fate by reprogramming cellular metabolism and activating survival pathways [290–293]. Notably, inhibition of the mitochondrial pyruvate carrier that reduces respiratory chain activity and ROS generation diminishes myofibroblast persistence in wounded corneas [294]. This situation also highlights mitochondrial ROS as critical regulators of apoptosis‐priming thresholds.

In summary, the relationship between ROS and myofibroblast apoptosis represents a dynamic tension. Contrary to the inherent roles of ROS in driving apoptosis, within the fibrotic niche, which is characterized by sustained TGF‐β signaling, ECM stiffness, and chronic OS, ROS predominantly facilitate the survival of myofibroblasts. This phenomenon overrides proapoptotic priming and stabilizes myofibroblasts. Therefore, the net effect of ROS is highly dependent on its sources, duration, magnitude, localization, and subcellular environment. Future research should investigate these subtle differences and identify specific ROS sources and molecules that can alter the balance toward apoptosis versus those that promote the survival of myofibroblasts.

### 6.4. ROS and Senescence‐Based Survival of Myofibroblasts

Traditionally, programmed cell death (apoptosis) was considered the primary mechanism for the disappearance of myofibroblasts. It is now evident that both senescence and apoptosis contribute to the elimination of myofibroblasts in recovering tissues, even within the cornea [295]. However, emerging evidence now indicates that senescent myofibroblasts are a group of persistent and pathologically significant cellular populations in fibrotic corneal complications, where they continue to influence tissue recovery through their altered secretory profiles and resistance to apoptosis [295–297].

Senescence is a state of permanent cell cycle arrest initiated by excessive stressors, which prevents the expansion of damaged cells [53, 298]. The arrested cell cycle is characterized by protein markers p16, p21, and p53, with senescent cells exhibiting enlarged, flattened morphology and positive senescence‐associated β‐galactosidase (SA‐β‐gal) staining [299, 300]. Under stressed conditions, cells undergo various metabolic and morphological changes to adopt a senescence‐associated secretory phenotype (SASP). This complex pro‐inflammatory state, primarily controlled by NF‐κB signaling [301, 302], fosters a local inflammatory microenvironment that enhances the phagocytosis of senescent cells by immune cells [303, 304]. The SASP comprises various cytokines, growth factors, MMPs, and ECM proteins such as IL‐1α, IL‐1β, IL‐6, IL‐8, TNF‐α, TGF‐β1, VEGF, PDGF, and IGF binding proteins (IGFBP)‐4 and ‐7, fibronectin, and collagens [52, 305–310].

Senescence critically regulates tissue homeostasis and is involved in both physiological and pathological processes, including development, repair, and remodeling, as well as disease progression [75, 311]. In the context of myofibroblasts, physiological senescence is transient and enhances tissue restitution through three mechanisms: (1) it accelerates wound closure by promoting myofibroblast differentiation via the deposition of specific SASP components, particularly PDGF [308]; (2) it regulates the population of proliferative and ECM‐depositing cells [70]; and (III) it resolves the stiff ECM through the activity of deposited MMPs [312]. These situations have been observed in suture‐injured corneas where senescent cells exhibited a nonfibrogenic phenotype, potentially aiding tissue restitution [295]. However, physiological senescence appears to follow spatiotemporal dynamics by generating an early fibrogenic SASP consisting of PDGF and ECM that persists for a few days, followed by a prolonged tissue‐remodeling SASP characterized by high MMPs expression.

Conversely, pathological senescence in myofibroblasts is permanent and promotes fibrosis and organ dysfunction. Senescent myofibroblasts with such characteristics, overspread their population through promoting senescence in neighboring cells (i.e., pro‐inflammatory immune cells and stromal cells) in a paracrine manner mediated by ROS‐induced DNA damage mechanisms and senescence‐associated ECM and cytokines, especially in the presence of TGF‐β [313–317]. These cells exhibit impaired capacity for dedifferentiation and demonstrate resistance to apoptosis, with excessive secretion of SASP fostering a local pro‐inflammatory and profibrotic environment [318–320]. As reported in alkali‐burnt corneas, senescent cells exhibited such fibrogenic characteristics and exacerbated the disaster [71].

Antioxidant treatments effectively inhibit senescence [321–325], highlighting the involvement of ROS in the induction of senescence. In fibroblasts/myofibroblasts, acquisition of SASP can occur through both DNA damage‐dependent and ‐independent pathways, such as p38 MAPK signaling. Physiologically, ROS triggers a nonfibrogenic senescence in myofibroblasts through p38 MAPK‐mediated p16 expression [326]. The p16, an inhibitor of CDK‐4 and ‐6, enables the conversion of these cells into scar‐resolving entities, causing a shift in their activities from ECM production to ECM resolution. Regulated ROS levels further enhance ECM resolution by upregulating MMP‐1 expression and enhancing the activities of MMP‐1, ‐2, ‐7, and ‐9 [212, 327–329]. Accordingly, senescent cells lose their cell–cell contacts and become more adherent to the ECM and resolve the peripheral matrix freely. While this state can persist indefinitely, chronic sublethal levels of ROS induce more genomic DNA damage, activating DDR and transitioning p16‐induced senescence into stress‐induced p53/p21‐dependent senescence arrest [298, 326, 330, 331].

Interestingly, excessive ROS overactivates p38 MAPK, which in turn promotes ATF‐2‐dependent overexpression of TGF‐β, including both latent and active forms. This ROS‐driven TGF‐β upregulation subsequently induces key senescence events, including morphological changes and the expression of senescence‐associated genes [332–334]. Therefore, by establishing a self‐reinforcing feedback loop, these events perpetuate TGF‐β and ROS levels, stabilizing senescence and further securing survival in stressed cells.

Additionally, ROS‐activated DDR disrupts mitochondrial permeability and respiratory coupling, gradually leading to complete mitochondrial dysfunction. In corneal myofibroblasts, mitochondrial dysfunction leads to the production of excessive amounts of ROS [294, 335], creating a feedback loop that perpetuates ROS levels and ROS‐induced DNA damage, stabilizing senescence in stressed cells. Accordingly, by maintaining antiapoptotic BCL‐2 family proteins (*e.g*., BCL‐2, BCL‐X_L_, and BCL‐W), mitochondria concomitantly secure apoptosis resistance in senescent myofibroblasts [55, 336, 337]. It has been reported that quercetin, a promising senolytic compound, binds directly to the BH3 domain of BCL‐2 and BCL‐X_L_ proteins, thereby inhibiting their activity and promoting apoptosis in senescent cells [324]. Thus, by preventing ROS‐mediated dysfunctional mitochondria, paracrine senescence expansion in myofibroblasts can be prevented through both ROS‐induced DDR [314, 338] and soluble SASP deposits [313].

In summary, senescence represents a double‐edged sword in the biology of myofibroblasts. While ROS‐induced p16‐mediated senescence in myofibroblasts promotes tissue restitution, late‐stage ROS‐induced p53‐dependent senescence is associated with their pathological sequelae. The latter amplifies mitochondrial dysfunction and SASP propagation that supports pathological myofibroblasts through paracrine senescence expansion and sustained profibrotic signaling. Thus, modulating ROS levels can balance the beneficial myofibroblasts and prevent their harmful perpetuation in recovery proceedings.

### 6.5. ROS and Autophagy‐Regulated Survival of Myofibroblasts

Autophagy is a dynamic cellular process in which objectionable intra and extracellular components are degraded by lysosomal machinery during cellular homeostasis. Experimental data indicate that dysregulated autophagy can lead to an increase in myofibroblast populations. It has been reported that utilizing drugs that regulate autophagy (e.g., rapamycin) maintains regular myofibroblast turnover [87]. Therefore, regulating autophagy can control myofibroblast population and improve corneal health.

Typically, autophagy is induced by various stressful stimuli such as hypoxia, starvation, and OS [339]. The process is mainly regulated by the two members of the phosphoinositide 3 (PI_3_)‐related kinase family: the Class III PI_3_ kinase and the mTOR kinase. Typically, the Class III PI_3_K controls the seeding of early autophagic structures, known as phagophores, whereas the mTOR prevents their formation [340].

ROS molecules regulate autophagy through the mTOR pathway [341], a pathway that conversely dampens ROS to homeostatic levels [342, 343]. The mTOR system consists of two distinct multiprotein complexes: Complex 1 (mTORC_1_) and Complex 2 (mTORC_2_). Regularly, mTORC1 maintains cellular energy and redox balance, whereas mTORC2 triggers adaptive survival pathways in response to stress or cellular damage [344]. Under prolonged ROS treatments, mTORC_1_ activation is reduced [345], resulting in the uncontrolled activation of mTORC_2_. Although not mechanistically interpreted, overactivation of mTORC_2_ indiscriminately drives senescence or myofibroblast transition in cells subjected to prolonged ROS exposure [72, 346–348]. Interestingly, in fibrotic situations, mTORC_2_ also acts as a positive regulator of mTORC_1_, creating a vicious cycle that ultimately promotes the deposition of ECM, especially collagen I [349]. Consequently, these ROS‐mediated events eventually lead to the accumulation of both functional and senescent myofibroblasts.

At sustained sublethal levels, ROS also impair the lysosomal activities of the autophagic machinery, leading to a progressive accumulation of oxidative damage [350, 351]. This mode of lysosomal impairment leads to the overaccumulation of dysfunctional cytoplasmic proteins and organelles, creating a state identical to cellular senescence, which is characterized by progressive cellular enlargement [352, 353]. Furthermore, impaired autophagic flux leads to cytosolic accumulation of dysfunctional mitochondria and aggregated proteins, resulting in chronically elevated intracellular ROS levels [354] and subsequent dysregulation of myofibroblast transition and development events such as α‐SMA expression and ECM deposition [67].

Interestingly, when damaged components cannot be degraded in autophagy lysosomes (i.e., autolysosomes), the autophagy machinery activates an unconventional secretion pathway to eliminate these components. Secretion through this pathway results in the misexpression of various inflammatory mediators and fibrogenic factors such as IL‐1β [355] and TGF‐β1 [356], suggesting an additional window for misinstructing myofibroblast differentiation and regulation [73, 357, 358]. Additionally, ROS‐compromised lysosomes release excessive levels of proteolytic enzymes that promote corneal thinning [359]. These enzymatic alterations create an ECM environment conducive to myofibroblast persistence.

Together, these ROS‐mediated disruptions of autophagy negatively impact both myofibroblast physiology and corneal recovery, highlighting the therapeutic potential of targeting ROS to mitigate their pathological sequelae. Based on reports, autophagy components are predominantly expressed in various parts of the eye, including the cornea [74], and this process is involved in multiple ocular complications [74, 360], including corneal scarring [87].

### 6.6. ROS and Immune–Based Survival of Myofibroblasts

Myofibroblasts can also persist through active evasion of immune clearance scenarios. Based on reports, it appears that their persistence is not merely a passive failure of apoptosis but an active process of immune evasion, where myofibroblasts employ multiple interconnected mechanisms to evade host immune surveillance [361].

Another key stage in corneal recovery is the coordinated infiltration of immune cells into the corneal stroma, which is orchestrated by the release of DAMPs and cytokines, including IL‐1, IL‐6, and TNF‐α, from damaged epithelial and stromal cells. This immune response occurs in a temporal sequence: neutrophils infiltrate within the first 24–48 h, followed by macrophages (which peak at 2–3 days) and NK cells (which are activated within 24–72 h). During resolution, various immune cells eliminate myofibroblasts through coordinated mechanisms, including TRAIL‐ and FasL‐mediated apoptosis by NK cells and CD8+ T cells, followed by efferocytosis by tissue‐resident macrophages [362, 363].

Although reestablishment of structural barriers normally terminates immune responses by interrupting immune cell infiltration, myofibroblasts in fibrotic corneas actively subvert this surveillance. Both regular and senescent myofibroblasts employ sophisticated strategies to evade immune clearance, with ROS playing crucial roles in their acquisition of immune privilege. This transformation reprograms physiologically transient repair cells into persistent pathological entities through interconnected molecular mechanisms.

While ROS actively regulate inflammatory cell influx (e.g., neutrophils and macrophages) and myofibroblast differentiation during recovery, they potentially protect myofibroblasts to fulfill their duties. Once established, ROS, in concert with TGF‐β, create an immunosuppressive microenvironment by disrupting antigen presentation pathways. They downregulate major histocompatibility complex class I (MHC‐I) molecules on myofibroblasts by inhibiting IFN‐γ‐dependent JAK/STAT1 signaling through oxidative modifications. Concurrently, ROS‐enhanced TGF‐β signaling suppresses MHC‐II expression by inhibiting CIITA (class II trans‐activator), the master regulator of MHC‐II transcription. Although it remained elusive in the cornea, this dual suppression of antigen presentation renders myofibroblasts effectively “invisible” to both CD8+ and CD4+ T cell surveillance [364–368].

Furthermore, ROS reduce the expression of costimulatory molecules (CD80/CD86) on antigen‐presenting cells while promoting T cell anergy through the direct oxidation of T cell receptors (TCRs) and the disruption of calcium signaling pathways, thereby impairing T cell activation and costimulation. This situation creates a state of immune tolerance where even if antigens are presented, the necessary secondary signals for T cell activation are absent [369–371].

ROS also orchestrate a profound shift in the local cytokine environment, favoring immunosuppression and fibrosis over immune activation. They elevate production of immunosuppressive cytokines, including TGF‐β, IL‐10, and IL‐13, while simultaneously suppressing immunostimulatory mediators such as IL‐12, TNF‐α, and IFN‐γ [372–379]. This cytokine imbalance promotes the recruitment of regulatory T cells (Tregs) through upregulation of chemokines, such as CCL17 and CCL22, further dampening effector T cell responses and establishing a tolerogenic microenvironment [379–382].

Additionally, specific ROS‐mediated signaling mechanisms, such as TRPV1 activation, promote the autocrine release of pro‐inflammatory cytokines, such as IL‐6, through p38 MAPK signaling by myofibroblasts, contributing to the immunosuppressive cytokine milieu that protects myofibroblasts from immune clearance [383, 384]. Within this milieu, the released IL‐6 mediates context‐specific functions that vary from its classical pro‐inflammatory effects, which typically occur in concert with cytokines like TNF‐α and IL‐1β to trigger an early, acute inflammatory response. For example, the released IL‐6 acts synergistically with other dominant cytokines in the milieu, such as TGF‐β and IL‐10. This synergy promotes the polarization of regulatory T cells—shifting CD4^+^ T cells toward a Th17 rather than a Th1 phenotype—and the differentiation and recruitment of myeloid‐derived suppressor cells (MDSCs), which collectively suppress cytotoxic immune surveillance by NK cells and cytotoxic T cells [385, 386].

Another critical ROS‐mediated immune evasion mechanism employed by myofibroblasts involves the upregulation of immune checkpoint proteins, such as programmed death‐ligand 1 (PD‐L1). ROS can directly induce PD‐L1 expression on myofibroblast surfaces by stabilizing hypoxia‐inducible factor‐1α (HIF‐1α) [387], a necessary component of hypoxia preconditioning protection employed by the cornea during recovery proceedings. When PD‐L1 engages with PD‐1 receptors on T cells, it delivers potent inhibitory signals that trigger T cell exhaustion or apoptosis. This interaction effectively converts potentially antifibrotic T cells into profibrotic effectors, creating a self‐reinforcing cycle of immune suppression and fibrosis promotion [238, 388–390].

Through this ROS‐mediated mechanism, a self‐perpetuating cycle is also created that maintains and amplifies myofibroblast persistence. ROS not only initiate myofibroblast differentiation by reducing the activity of HIF‐prolyl‐hydroxylases (PHDs) and HIF‐1α stabilization but also enhance their immune evasion capabilities through multiple pathways. The ROS‐HIF‐1α axis upregulates chemokines such as CXCL12, which recruit additional immunosuppressive cells and create barriers to immune surveillance [391, 392].

Manipulation of death receptor pathways, such as the Fas/FasL system, is another mechanism by which myofibroblasts evade immune responses. While immune‐evaded myofibroblasts often overexpress functional Fas Ligand (FasL) compared to regular myofibroblasts, they paradoxically become highly resistant to Fas‐induced apoptosis. This resistance operates through multiple mechanisms, including reduced surface Fas expression due to ROS exposure, ROS‐mediated increased matrix stiffness, elevated production of soluble Fas (sFas), and the production of decoy receptors like DcR3, which sequester death ligands and inhibit T cell/NK‐cell‐induced extrinsic apoptosis [393–395].

Beyond resistance to death signals, myofibroblasts weaponize FasL expression to launch counterattacks against approaching immune cells. By expressing functional FasL on their surface, myofibroblasts can induce apoptosis in Fas‐expressing immune cells, particularly CD8+ T cells [238]. This situation allows myofibroblasts to eliminate immune cells that would otherwise target them for destruction, creating an immune‐privileged environment more conducive to their persistence. This FasL‐mediated killing also reprograms surviving CD4+ T cells, converting them from antifibrotic to profibrotic phenotypes that secrete IL‐17A and TGF‐β [238], establishing another vicious, self‐amplifying feedback loop that perpetuates both fibrosis and immune suppression.

As established, senescent myofibroblasts represent another pathogenic population of myofibroblasts that combines growth arrest with enhanced immune evasion capabilities. These cells adopt a SASP, releasing inflammatory cytokines, chemokines, and growth factors that paradoxically can promote their survival while suppressing immune clearance.

To evade phagocytic clearance, senescent myofibroblasts employ distinct characteristics of SASP. The SASP includes factors such as IL‐6, IL‐8, and PAI‐1, which block caspase activation and promote Bcl‐2 expression, thereby enhancing the survival of senescent cells. Additionally, the SASP release also includes TGF‐β and IL‐10, which recruit M2 macrophages and Tregs, thereby suppressing NK‐cell activity. By releasing CXCR2 ligands, such as IL‐8, SASP also blocks NK‐cell chemotaxis into fibrotic foci, creating additional barriers to immune elimination [299, 396–400].

Critically, ROS also upregulate “do not eat me” signals on senescent myofibroblasts, particularly CD47 [401–403]. As CD47 binds to SIRPα receptors on phagocytes, it actively inhibits engulfment and allows senescent cells to persist despite expressing “eat me” signals. This mechanism is particularly important in corneal fibrosis, where efficient efferocytosis is essential for tissue homeostasis [404, 405].

Senescent myofibroblasts also exhibit increased expression of immune checkpoint molecules beyond PD‐L1, including PD‐L2, which engages PD‐1 receptors on NK cells and T cells to induce exhaustion [406–408]. They also upregulate HLA‐E, a nonclassical MHC class I molecule that engages NKG2A receptors on NK cells and highly differentiated CD8+ T cells, thereby providing an additional layer of immune protection [409]. This ROS‐mediated upregulation of PD‐L1 and HLA molecules occurs primarily through the activation of the p38 MAPK signaling pathway and the release of SASP factors, such as IL‐6, both of which are strongly influenced by ROS [410, 411].

The interplay between ROS and increased ECM stiffness creates additional barriers to immune clearance. The dense, disorganized ECM deposited by myofibroblasts creates both physical and biochemical barriers to immune clearance. ROS‐mediated cross‐linking increases matrix stiffness, which mechanically inhibits immune cell infiltration and creates protected niches for myofibroblast persistence.

Increased matrix stiffness also has direct effects on immune checkpoint expression, amplifying PD‐L1 levels on myofibroblasts through mechano‐transduction pathways. This stiffness‐PD‐L1 axis creates a self‐reinforcing mechanism where matrix deposition enhances immune evasion, allowing further matrix accumulation [238, 395, 412, 413].

Synergistically, the corneal‐specific microenvironment provides additional layers of immune privilege that complement and enhance ROS‐mediated evasion mechanisms. Primarily, the aqueous humor bathes the cornea in high concentrations of immunosuppressive factors, particularly TGF‐β2, creating a constitutively immunosuppressive environment [414]. Concomitantly, in pathological conditions, the anterior chamber‐associated immune deviation (ACAID) inadvertently leads to the persistence of myofibroblasts through systemic modulation of immunoregulatory responses, characterized by increased TGF‐β and IL‐10 levels and decreased IFN‐γ levels. This chamber establishes the cornea’s physiological tolerance to antigens by selectively suppressing delayed‐type hypersensitivity while preserving humoral immunity [415, 416]. Additionally, this privilege is reinforced by the cornea’s limited lymphatic drainage, which restricts antigen trafficking and delays adaptive immune responses. In combination, these features provide myofibroblasts additional time to establish their pathological characteristics.

Together, the complexity of immune evasion strategies employed by myofibroblasts underscores the need for multitargeted therapeutic approaches. Therefore, as ROS effectively mediate these strategies, future therapeutics should focus on disrupting ROS‐mediated self‐reinforcing pathological loops to restore regular immune surveillance and clearance in the corneal microenvironment.

### 6.7. ROS and Structural Reintegration–Based Survival of Myofibroblasts

As previously noted, the corneal epithelium and endothelium, along with their supportive membranes, regulate molecular transport between the stromal and extra‐stromal compartments [1]. Consequently, the delayed or failed reintegration of these layers creates an opening for the free diffusion of biomolecules into the injured stroma, where these factors can alter the physiology of myofibroblasts [417]. Essential extracellular requisites for myofibroblasts’ persistence, including profibrotic factors (e.g., TGF‐β) and extracellular vesicles, readily infiltrate into the stroma when these layers are compromised [76, 418]. For instance, a postphotorefractive keratectomy study revealed that TGF‐β diffusion across corneal structures is a primary survival mechanism for stromal myofibroblasts [49]. This suggests that impaired structural reintegration enables myofibroblasts to maintain access to essential survival factors. Consistent with this observation, stromal myofibroblasts prefer to accumulate near damaged areas of both anterior and posterior stromal borders [58, 419]. Intriguingly, myofibroblasts may even actively prevent structural reintegration through the paracrine induction of apoptosis in boundary‐forming cells [120], thereby maintaining their access to the surrounding TGF‐β‐rich aqueous humor.

Primarily, the stromal barrier cells (i.e., epithelial and endothelial cells) exhibit high susceptibility to ROS‐mediated apoptosis [120, 420, 421]. Additionally, ROS disrupt the barrier function of these layers by affecting the interaction between the components of tight/adherent junctions (e.g., occludin and Zonula occludens proteins) between cells that furnish these layers [422]. Simultaneously, these molecules can oxidize key components of the basement membranes, such as laminin and fibronectin. Oxidation of cysteine residues in these proteins disrupts their tertiary structure and ligand‐binding capacity, leading to weakened cellular‐basement membrane adhesion. In addition, ROS can impair hemidesmosome formation by downregulating integrin α6β4, a key laminin receptor essential for cellular‐basement membrane anchoring [423].

**Figure 6:**
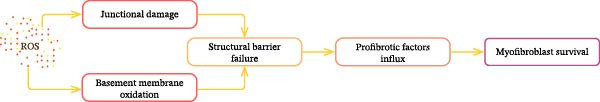
Schematic representation of ROS‐mediated structural irregularities and myofibroblast survival.

Together, as illustrated in Figure 6, ROS make corneal layers vulnerable to paracellular permeation. These unintentional structural permeabilities create microenvironments where myofibroblasts maintain access to critical survival factors freely, eventually disrupting their physiological equilibrium of formation and apoptosis.

## 7. Therapeutic Implications and Strategies

Based on the intricate ROS and myofibroblast interplay in corneal pathology, potential therapeutic targets can include (1) TGF‐β signaling, (2) ECM composition and remodeling factors, (3) autophagy regulators, and (4) mediators of mitochondrial dysfunction. However, due to mechanistic difficulty in identifying the culprit mechanism (s) in this network, directly targeting ROS may represent a more strategic approach. As upstream mediators of multiple pathomechanisms, ROS suppression could simultaneously mitigate several detrimental processes, thereby offering broader therapeutic efficacy.

To date, ROS‐controlling therapeutic interventions for mitigating myofibroblast‐related complications in the cornea remain experimental. However, researchers have explored several strategies to elucidate their potential for future clinical translation. The majority of these approaches aimed to rebalance the fibroblast‐myofibroblast equilibrium after insults, as pathological shifts in this ratio allow detrimental sequelae of myofibroblasts to prevail over the restorative capacities of fibroblasts, ultimately leading to imperfect tissue restitution. Restoring this physiological balance could change the situation in favor of the tissue restitution machinery, allowing it to effectively regain its potential and restore the natural environment as intended.

ROS‐modulating therapeutic approaches have targeted both direct inhibition of ROS generation and indirect neutralization of their downstream effects. In line with the first approach, several studies indicated that the suppression of ROS effectively prevents myofibroblast differentiation. It has been reported that pharmacological inhibition of NOX enzymes (e.g., diphenyleneiodonium) has prevented the formation of ROS and myofibroblast development [143]. Alternatively, administration of enzymatic or nonenzymatic antioxidants such as SOD [424] and NAC (229), respectively, has shown comparable efficacy and results. Notably, enhancing endogenous antioxidant responses through augmented NRF2 pathway activation similarly inhibits myofibroblast formation [167, 425]. In a complementary approach, other competitive mechanisms, besides those induced by ROS, have also been targeted. For example, by utilizing sodium nitrite as a NO donor, the inhibitory role of the NO/cGMP pathway is enhanced, effectively counteracting TGF‐β1/ROS‐mediated myofibroblast differentiation, as demonstrated in both in vitro and in vivo models [426].

## 8. Conclusion and Future Remarks

Corneal opacification and fibrosis visually impair millions worldwide [427, 428], leaving corneal replacement the ultimate solution for most cases. Myofibroblasts, as critical components of the tissue reconstruction machinery, bear the main culprits in forming tissue disharmonies following corneal insults, including infections, traumas, and surgeries. They establish such pathological sequels through their overpresence and overactivity that exceed physiological boundaries. Unfortunately, the physiology of these fibrogenic cells can be negatively influenced by various cellular and environmental factors and events, making it challenging to avoid their pathogenicity in recovering tissues.

ROS molecules, as an important group of signaling molecules, can negatively impact corneal restitution by modulating the physiology of myofibroblasts. Excessive production of ROS, patronaged by diminished levels of antioxidants, leads to individually or collectively enhanced secretion and activation of growth factors, sustained deposition and modification of the ECM components, apoptosis‐resistance reprogramming, and acquisition of pathological phenotypes. Therefore, annihilating ROS overaccumulation appears promising as an attractive prescarring therapeutic strategy pertinent to pathological myofibroblasts. This strategy is plausible since ROS can drive multiple pathomechanisms concomitantly; therefore, their suppression would prevent multiple disasters simultaneously. Although, to fully benefit from such intervention, a few considerations must be taken into account, including; (1) an estimation of the time of proper intervention (based on routine follow‐ups of the outcome of the tissue’s restitution machinery), as tissue reconstruction is highly dependent on physiological levels of ROS [241] or (2) an enhanced delivery strategy for pharmacological therapies, as systemic interventions are not ideal choices for corneal disasters [429].

## 9. Caveats and Limitations

The findings discussed here primarily rely on in vitro and ex vivo data, which may not fully resemble the in vivo systems. While these models provide valuable insights, the in vivo expansion of these findings, even those performed on extracted biopsies, requires further verification, especially considering the dynamic interplay of factors such as immune responses and neural regulation in recovering tissues.

ROS also present a unique challenge to this study. As transient signaling molecules, ROS should only be considered pathological when they exist at elevated levels for extended periods. However, demonstrating this situation in vivo is inherently difficult due to the short‐lived nature of these events and the lack of proper tools for continuous monitoring in intact biological systems.

Moreover, most evidence presented here for ROS‐mediated pathology derives from controlled ex vivo environments. While these studies likely reflect the regulatory potential of ROS at perturbed levels, they cannot fully replicate the ROS perpetuations that occur during tissue recovery stages.

## Funding

No funding was received for this manuscript.

## Conflicts of Interest

The authors declare no conflicts of interest.

## Data Availability

Data sharing is not applicable to this article as no datasets were generated or analyzed during the current study.
